# Drug-Eluting Nasal Implants: Formulation, Characterization, Clinical Applications and Challenges

**DOI:** 10.3390/pharmaceutics6020249

**Published:** 2014-05-27

**Authors:** Ankit Parikh, Utkarshini Anand, Malachy C. Ugwu, Tiam Feridooni, Emad Massoud, Remigius U. Agu

**Affiliations:** 1 Biopharmaceutics and Drug Delivery Lab, College of Pharmacy, 5968 College Street, P.O. Box 15000, Halifax, NS B3H 4R2, Canada; E-Mails: AN347441@dal.ca (A.P.); U.Anand@dal.ca (U.A.); ugwumcc@yahoo.com (M.C.U.); tiam.feridooni@gmail.com (T.F.); 2 Queen Elizabeth II (QEII) Health Sciences Centre, 1278 Tower Road, Halifax, NS B3H 2Y9, Canada; E-Mail: Emad.massoud@dal.ca

**Keywords:** nasal, sinuses, rhinosinusitis, stents, implants, inserts, sustained-release

## Abstract

Chronic inflammation and infection of the nasal sinuses, also referred to as Chronic Rhinosinusitis (CRS), severely affects patients’ quality of life. Adhesions, ostial stenosis, infection and inflammation relapses complicate chronic sinusitis treatment strategies. Drug-eluting stents, packings or implants have been suggested as reasonable alternatives for addressing these concerns. This article reviewed potential drug candidates for nasal implants, formulation methods/optimization and characterization methods. Clinical applications and important considerations were also addressed. Clinically-approved implants (Propel™ implant, the Relieva stratus™ MicroFlow spacer, and the Sinu-Foam™ spacer) for CRS treatment was an important focus. The advantages and limitations, as well as future considerations, challenges and the need for additional research in the field of nasal drug implant development, were discussed.

## 1. Introduction

The nose plays a crucial role in airway homeostasis by warming up, humidifying and filtering incoming air. This function may not be possible without the paranasal sinuses. The sinuses, especially the maxillary sinuses are prone to diseases and chronic inflammation. Prior to exploring drug delivery strategies to the sinuses and its challenges, it is important to discuss the anatomy and physiology of the nose and paranasal sinuses, as well as chronic sinusitis, the commonest chronic disease that affects this area.

### 1.1. Anatomy and Physiology of the Nose and Paranasal Sinuses

**Figure 1:**
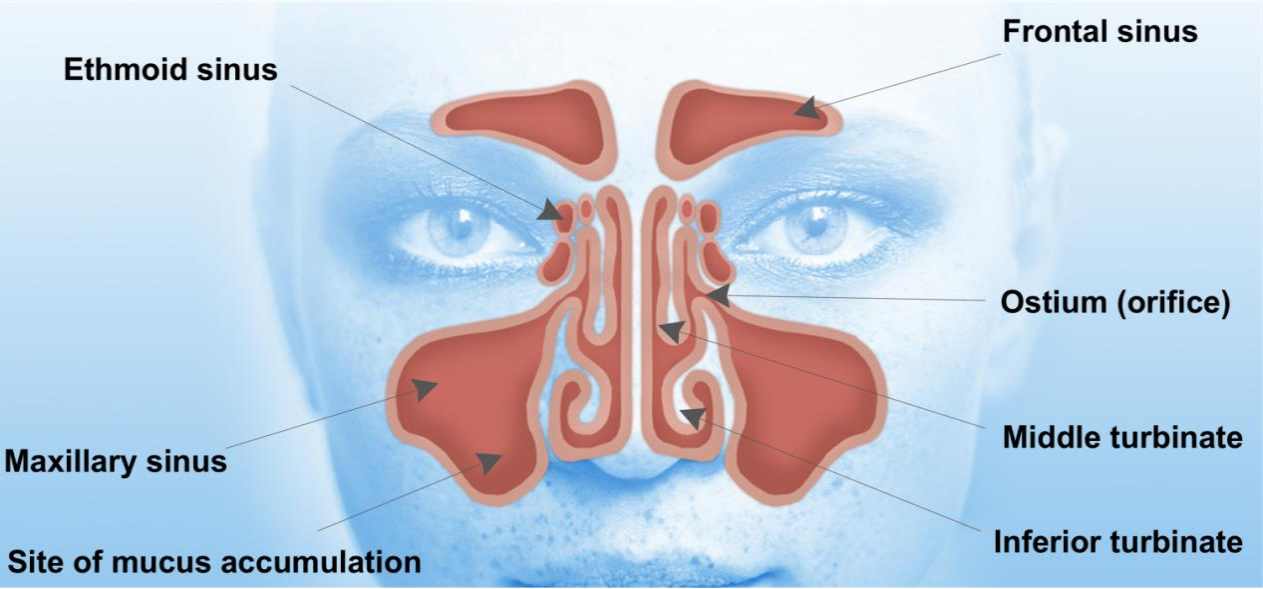
Human facial anatomy showing the location of sinuses. Entrance to the maxillary sinuses can be blocked by polyps and chronic inflammation, making it impossible for nasal sprays to penetrate the maxillary sinuses.

The paranasal sinuses are air-filled cavities found in the facial bones [1]. These cavities are connected to the nasal cavity to form a complex system at the entrance of the upper airway (Figure 1). This complex unit has highly specific functions, which include humidifying, filtering, warming and air conditioning of the inhaled air to form an immunological response against particles in the inspired air, thereby protecting the delicate structures of the lower respiratory system [2,3]. The paranasal sinuses include frontal, maxillary, ethmoid and sphenoid sinuses [3]. The maxillary sinuses are the largest of the sinuses and are located in the cheek, whereas the ethmoid sinuses are located in the anterior base of the skull [2,4]. Frontal and sphenoid sinuses are located in the frontal (forehead) [4] and sphenoid (skull base) bones, respectively [2,4]. The nasal cavity opens anteriorly in the nostrils and connects posteriorly to the nasopharynx. The lateral wall of the nasal cavity is formed by the surfaces of the lacrimal bones and the maxillae and supports the inferior, middle and superior turbinates [2,5]. These turbinates divide the nasal cavity into the inferior, middle and superior meatus. The middle meatus contains the orifices of the frontal, maxillary and the anterior cells of the ethmoid sinuses. These sinuses drain into the osteomeatal complex. The blockage of the ostium results in inflammation, especially within the maxillary sinuses leading to mucosal swelling and accumulation of secretions.

Inflammation in the paranasal sinuses is often associated with inflammation within the nasal cavity hence the term chronic rhinosinusitis (CRS) is sometimes used to describe the condition. CRS is a clinical syndrome associated with persistent inflammation of the nasal mucosa and paranasal sinuses which encompasses both polypoid (CRSwNP) and non-polypoid (CRSsNP) forms of the disease [6,7,8]. Chronic inflammation of the sinuses results in nasal obstruction, thick nasal discharge, reduction or loss of olfaction and facial pressure or pain [9]. The pathophysiology of CRS is characterized by a diversity of immunological mechanisms involving the T-cells, eosinophilic/neutrophilic inflammation, and airway remodeling. Therefore, CRS with polyps has been specifically associated with asthma and cystic fibrosis [10]. Some CRS patients may require functional endoscopic sinus surgery (FESS) to resolve the condition. Drug-eluting nasal stents/implants can be used as adjuncts to endoscopic sinus surgery to enable better sinus drainage and wound healing.

### 1.2. Current Strategies to Deliver Drugs to the Nasal Sinuses and the Need for Improvement

Management of CRS can be complex, and definitive evidence-based protocols are not currently defined because of the disease heterogeneity, incomplete understanding of its refractory characteristics and differences in individual responses to various interventions [11]. Currently, the initial treatment for uncomplicated CRS is conservative medical therapy, including antibiotics and corticosteroids [12]. Functional endoscopic sinus surgery is widely considered to be the standard treatment for medically refractory CRS [7]. Success in outcomes of FESS in patients with CRS with polyps is heavily dependent on reducing postoperative scarring, edema, and crusting that can inhibit natural ciliary function and sinus drainage [13]. Many CRS patients with polyps do not respond well to inhaled steroids and the polyps often re-grow following surgery [1]. Therefore, treatment of CRS with polyps is still an unmet medical need. Also, CRS arises from prolonged obstruction of the osteomeatal complex, thus leading to mucociliary dysfunction preventing mucous drainage, and failure to clear bacteria from the sinuses [14]. Many drugs used for treating chronic sinusitis are given as nasal sprays or oral formulations. Though, various methods of topical drug delivery such as nasal drops and nasal sprays are generally well accepted, only a few studies have concentrated on intranasal drug distribution [14]. Unfortunately, sprays fail to target potentially infected anatomic sites such as the maxillary sinus, ethmoid cells or middle turbinate because these areas are occluded from the nasal passage due to inflammation [15]. As CRS is a condition which lasts for duration longer than 12 weeks, a drug delivery system with prolonged mucosal contact time with local absorption and minimal depletion are often the desired requirements [14,16]. A variety of adjunctive devices have been applied to the sinuses during functional endoscopic sinus surgery (FESS) to keep the middle meatus open, with varying success; these include packing materials, injectable space-filling gels or structured stents [17]. Recent studies have shown that soaking these packing materials with drugs during surgery showed inconsistent results in terms of wound healing, maintenance of ostium patency and prevention of polyposis recurrence. Moreover, drug release from nasal packing materials is uncontrolled and inconsistent which may explain the erratic outcome of this treatment strategy. For these reasons, nasal drug-eluting implants with prolonged mucosal contact time which releases the drug locally to the affected site for a prolonged period of time appear to be an option that may assist in solving some of these problems.

### 1.3. Drug Eluting Stents and Implants: Definition and Nomenclature

**Figure 2:**
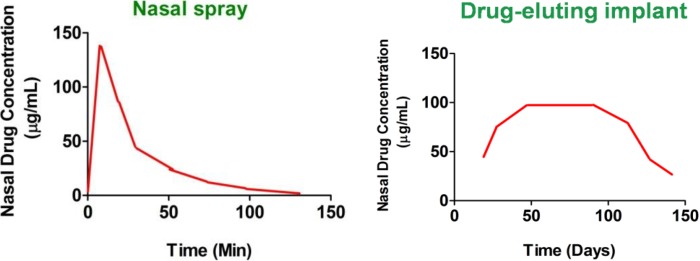
Comparison of the plots of nasal drug concentration *versus* time, obtained after administration of nasal sprays and drug-eluting implants. Nasal sprays show rapid clearance of the drug from the nasal mucosa as compared to locally acting implants.

Stent is defined as a device which is placed into a cavity temporarily to keep it open, promote wound healing and relieve an obstruction [18], whereas FDA defines implant as a device which can be placed in to a naturally or surgically formed cavity of the human body in order to remain there for a period of 30 days or more. However, in order to protect public health and depending on the intended application, FDA may also determine that devices placed for shorter periods are also implants [19]. Drug eluting stents (DESs) or implants are surgically inserted scaffolds that help in healing the affected tissue by releasing loaded-drug locally and continuously in a controlled manner for the desired period of time [20]. Thus, a stent or an implant is basically a support placed temporarily inside a cavity, duct or a blood vessel to aid healing and/or relieve an obstruction [21]. However, in the context of this review paper, “implant” is a more suitable term to describe drug-eluting nasal devices as CRS being a chronic condition requires the medical devices to be implanted for duration greater than 30 days for prolonged drug release. Implants, in general have a wide range of applications, and are used to improve the quality of function, and hence the quality of life of the people who use them [22]. Some examples are hip, dental, cochlear, neural, spinal, retinal and nasal implants [22,23,24,25,26]. Nasal implants are devices that are inserted in the nose following nasal or paranasal sinus surgery. These drug-eluting devices release drug-loads slowly and continuously from polymer matrices to affected areas in the sinuses or nasal cavities for a prolonged period of time. The devices may be used to locally treat nasal and paranasal infections, inflammations, neoplasm, autoimmune diseases and nasal reconstruction for aesthetic deformities [27]. Nasal implants can also be effectively used for the treatment of sinusitis [28]. In this paper implants are used to describe both drug-loaded and non-loaded devices used to facilitate wound healing and prevention of polyps re-occurrence following FESS. The major advantage of nasal drug-eluting implants compared to standard nasal sprays is summarized in Figure 2. Following standard nasal sprays, administered drugs are removed within a few days by the mucociliary clearance. Little or no drugs are detected within a few hours (nasal sprays). In contrast, drug-eluting nasal implants ensure continuous drug release over prolonged period of time to the affected mucosa for CRS treatment (drug-eluting implant). Therefore, nasal sprays that are currently used for CRS treatment [29] not only fail to target the potentially affected anatomic sites [15] but also have short duration of action compared to drug-eluting stents.

## 2. Formulation and Development of Nasal Implants

### 2.1. Development of Implants and the Need for Biodegradable/Bioabsorbable Impants

Most of the knowledge that are currently applied to nasal implants development were based on data acquired from cardiac stents. Bare metal stents (non-drug eluting) were the first of the approved stents. Due to their primitive design, they are rarely used owing to the complications arising from their use. Metallic stents are known to cause stent thrombosis that requires prolonged antiplatelet therapy. Due to their rigid structure, they also prevent lumen expansion [30,31]. An important breakthrough in stents research is the development of drug-eluting stents (DES) [31]. Although some drug-eluting stents have a metallic stent backbone, they are coated with a polymer that serves as a vehicle for the drug and elutes the drug load in a controlled manner to the surrounding affected areas [32]. Even though drug-eluting metal stents significantly reduce the rate of restenosis, there are certain limitations associated with these stents. They cause late stent thrombosis and may also cause chronic inflammation at the stent site [31,33]. Advances in stents research have led to the development of biodegradable stents and implants. Drug-eluting implants and stents can be biodegradable/bioabsorbable or non-biodegradable (metallic stents). These biodegradable drug-eluting devices are preferred over metal stents as they do not cause late stent thrombosis due to their bioabsorbable nature. Biodegradable implants are made up of biodegradable polymeric materials that degrade *in vivo* over a prolonged period of time. The major advantage of these implants is that no additional surgeries are required to remove them [31].

### 2.2. Formulation Considerations, Biodegradable Materials and Drug Candidates for Formulating Nasal Drug-Eluting Stents

**Figure 3:**
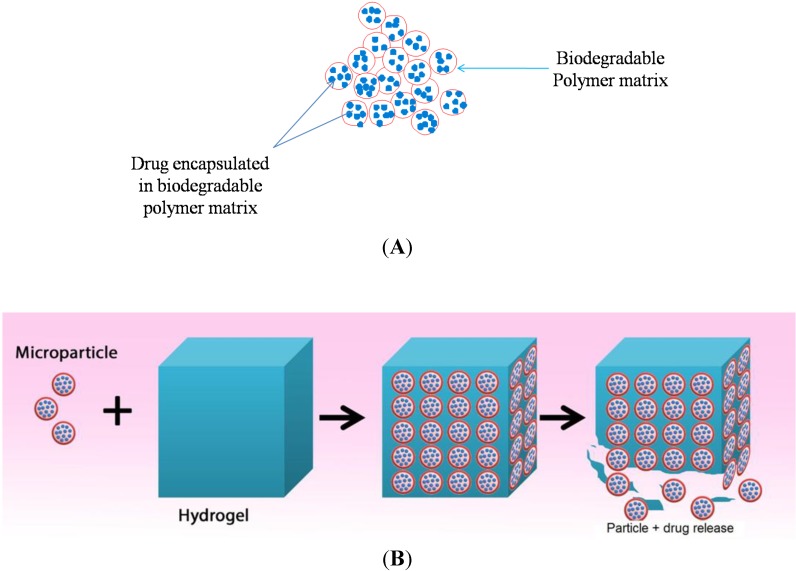
(**A**) Drug encapsulated in a biodegradable polymer matrix; and (**B**) Drug loaded microparticles are incorporated in a hydrogel to form an implant, which degrades in a controlled manner to release the microparticles.

In chronic nasal conditions such as CRS, infection and inflammation becomes persistent and lasts for a very long duration of time (>12 weeks) [16]. Thus, it is necessary to achieve a sustained drug release over a long period of time (>2 months). This can be achieved by drug encapsulation in a biodegradable polymer matrix in form of micro or nano particles (Figure 3A) [34].

**Figure 4:**
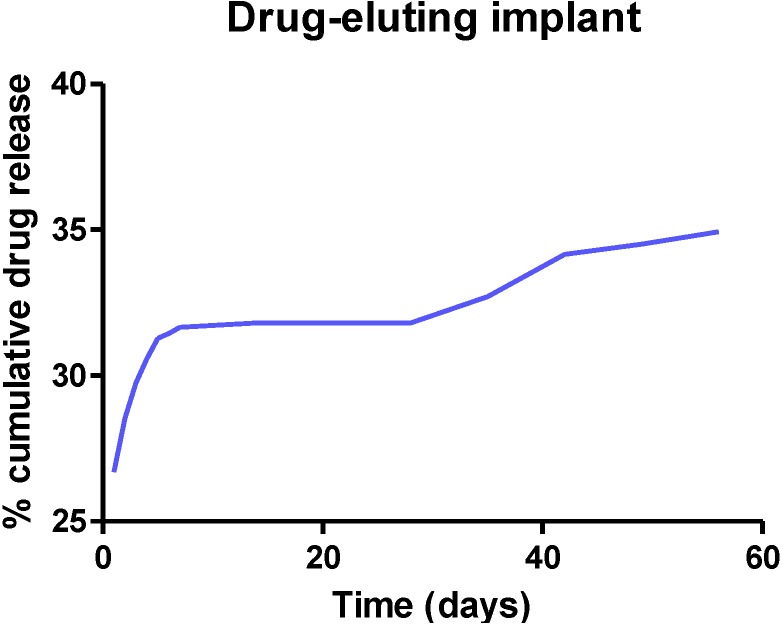
A plot of percentage cumulative drug release from the implant *versus* time shows the initial burst release of the drug and then releases the drug slowly and continuously for longer than two months.

These micro or nano particles can then be incorporated in a biodegradable hydrogel to form a biodegradable implant [35]. The hydrogel degrades in a controlled fashion to release the drug-loaded microparticles (Figure 3B), which in turn degrades slowly and consistently over a period of time to release loaded drug. Ideally, biodegradable implants should release loaded drugs over a long period of time as shown in Figure 4.

Biodegradable polymers such as polylactic acid (PLA) [36,37] or polylactic-co-glycolic acid (PLGA) [38] can be used effectively for nasal implants as they have a long history of safety and effectiveness in humans [39]. PLA and PLGA are also used extensively for a variety of pharmaceutical applications, drug delivery devices and scaffolds fabrication for tissue engineering [40]. Other biodegradable polymers that can be used for nasal implants fabrication include complex sugars such as alginates [41], hyaluronates [42,43] and chitosan [44].

As for drug candidates for nasal stents, the most commonly used drugs for nasal conditions include, but are not limited to: Corticosteroids such as dexamethasone, fluticasone and mometasone [45], as well as antibiotics for bacterial infections [45].

### 2.3. Formulation Methods for Biodegradable Nasal Implants

Several methods and strategies may be employed for the preparation of drug-eluting nasal implants. Main methods include solvent casting, solvent extrusion and compression. In solvent casting method, extended release polymers are dissolved in a suitable solvent such dichloromethane [46] or acetone [38]. Sterile drug solution is then added to the polymer solution under gentle stirring. The solution is then allowed to settle for a few minutes and then cast in to a teflon mold of a desired diameter. The films are allowed to dry for a few days or vacuum dried to completely remove solvent residues and then cut in to pieces of desired size [46,47]. An example of this type of implant is dexamethasone/ethyl vinyl acetate (EVAC) scaffold. Although this approach is not based on micro or nano particles approach, the drug is entrapped in EVAC polymer matrix that enables a constant release of dexamethasone for over 30 days [46,47]. Solvent casting methods have several disadvantages and cannot be used for industrial scale up as they require large amounts of organic solvents to dissolve PLGA and drugs to fabricate into an implant. It is also necessary to allow the organic solvent to evaporate completely from the implant, which takes a very long time [38,48]. Extrusion is another method for implant development [38,47]. This method can be used with or without organic solvents [47]. For solvent-associated extrusion method, the polymer and drug are dissolved in a suitable volatile organic solvent. This solution is then extruded through a small orifice (syringe and a silicone tube can be used for this purpose). The solvent is allowed to evaporate completely to form an extrudate. This method requires a high concentration of polymer and hence micronization may be required when drug powders are added to the high polymer concentrate [47]. Solvent-associated methods are not ideal because these methods involve the use of toxic organic solvents and drug stability may also be an issue. In the extrusion method devoid of solvents, polymer-drug powder mixture is heated to a semi-solid state using heating elements and shear stress from the extrusion screw. The screw pushes the drug/polymer mixture through the die to form an extrudate that cools and solidifies to form an implant of desired size [38,49]. Although this method does not require solvents, a major setback is its unsuitability for heat sensitive drugs. Compression molding can be used alone (direct compression) [47] or in combination with solvent casting method. The solvent cast drug/polymer composite can be compression-molded at a suitable temperature and pressure to desired shape [38,50,51]. Unlike casting and solvent extrusion methods, compression method is less stressful, suitable for drugs that are sensitive to heat, moisture or solvent and no solvents are used. Implants prepared by this method are highly porous and may sometimes show faster drug release and additional processing may be necessary to prolong drug release. Coating after compression may be useful to retard drug release [47].

### 2.4. Methods for Developing Nano/Microparticles/Polymer Composites for Drug-Eluting Implants

Post-surgical treatment of CRS requires prolonged and continuous drug release to the affected sinuses. If drugs are incorporated into a polymer matrix (e.g., packing sponge), there is faster rate of drug release and pharmacological effects are limited to a few days. The rate of drug release would slow down considerably if the drugs are formulated as biodegradable micro/nanoparticles that are then incorporated into a polymer matrix composite that can be cast into implants [52]. Thus, an alternative approach for making nasal implants is to formulate them as micro or nano particles using biodegradable and non-biodegradable polymers. The encapsulated drug is subsequently delivered using hydrogels or other types of scaffolds or composites. Some specific examples include microparticles/alginate scaffolds/composites, microparticles/polyvinyl alcohol (PVA) hydrogel, microparticles/hyaluronate scaffolds/composites and microparticles/collagen scaffolds/composites. The microparticle/hydrogel scaffold using alginate can be prepared using an ionic cross linking process. In this method, a 2% *w*/*v* alginate solution is added to suspension of microspheres followed by a 1% *w*/*v* calcium chloride solution, added as cross linking agent [41]. For microparticles/PVA hydrogel, drug-loaded biodegradable microparticles embedded in PVA matrix can be prepared by dispersing the microparticles in a 5%–10% aqueous solution of PVA (99% hydrolyzed, molecular weight: 133 kDa), followed by homogenization at a suitable speed. The microparticles/PVA dispersion is then applied to a suitable mold and subjected to a number of freeze-thaw cycles; by freezing at −20 °C (for 1–2 h) and thawing at room temperature (30 min to 1 h) [35,53,54]. The number of freeze-thaw cycles depends on the desired properties of the hydrogel and the release profile. Additives such dextran or humic acid can be added to the PVA solution to modify drug release profile as desired [53,54]. Unlike the two methods described above, microparticles/hyaluronate composites can be prepared by dispersing the microparticles in an aqueous solution of sodium hyaluronate. Adipic dihydrazide is then added with thorough stirring to increase the mechanical strength of the hydrogel. After adjusting the pH to the desired value, *N*,*N*-dimethylaminopropyl carbodiimide (EDC) is added as cross-linking agent [42]. Microparticles/collagen scaffolds/composites can be prepared by freeze drying method. In this method, collagen is dissolved in 3% acetic acid solution. Subsequently, dried collagen scaffold are then cross-linked with nordihydroguaiaretic acid (NDGA). The scaffolds are suspended in PBS and agitated in the NDGA solution for 24 h at room temperature. These scaffolds are then removed and freeze-dried [55].

## 3. Characterization of Drug-Eluting Nasal Implants

In order for nasal implants to work optimally, they must be optimized for morphological appearance, physicochemical characteristics, drug release, and *in vivo* performance in animal models. During morphological examination, the microstructure of the implants is examined by scanning electron microscopy (SEM) after gold [56] or platinum spluttering [57]. The morphological analysis provides useful information regarding sample composition. SEM analysis enables us to examine the cross-linking intensity and the porosity of the implant. Microscopy also makes it possible to explore the characteristic network structure of the implant [43,58].

For drug release studies, implants or composites are immersed in a suitable release medium comprising of phosphate-buffered saline (PBS) at pH 7.4. They are agitated at suitable speeds using a shaker incubator maintained at 37 °C. At pre-determined time points, aliquots are withdrawn (and replaced with equal volumes of the same release medium to maintain sink conditions) and analyzed for drug content using a suitable analytical technique such as HPLC. A plot of percentage cumulative drug release *versus* time is obtained to determine the *in vitro* drug release profile of the implant [35,52,55].

Animal studies provide useful information on *in vivo* drug release from implants, adverse effects (if any), and the effect of incorporated drug on the nasal histology. Animal studies make it possible to monitor inflammatory changes in the nasal and sinus mucosa over a period of time [59,60]. Rabbits are the most suitable animal model for evaluating nasal drug-eluting implants [59,60,61]. This is because the sinuses of rabbits and humans show similarities with respect to their anatomies [62]. The *in vivo* characterization of nasal implants includes histological and nasal lavage analyses, as well as peripheral blood levels screening [59]. During *in vivo* characterization, standard surgical procedures are performed and the implant is placed in the maxillary sinus [59,60]. As the sinuses are paired cavities, one of the sinuses receives the drug-loaded implant, whereas no implant is placed in the other sinus. A control group of animals receive non-drug eluting implant [60]. After surgical implantation, the animals are sacrificed at selected post-operative days for histological analysis following standard hematoxylin and eosin staining. Histological analysis of the sinus mucosa is used to determine mucosal, luminal and inflammatory changes due to the implants [59,61], as well as the extent of implant degradation [59]. In standard experiments, inflammatory changes in the group receiving drug-eluting implants are compared to the group receiving non-drug eluting implant [60]. In standard animal studies, drug levels are also analyzed in the nasal lavage and blood [59,60].

## 4. Clinical Applications of Drug-Eluting Nasal Implants

Incorporation of a drug such as corticosteroids, antibiotics or anti-neoplastic agents into nasal implants is the primary focus of developing drug-eluting nasal implants [63]. CRS is the primary medical condition for drug-eluting nasal implants. As nasal obstruction is the most common symptom of CRS [64], the aim of its treatment is to restore sinus ventilation, promote mucous drainage and reduce edema [12,65]. Although the first line of treatment for a patient with CRS is conservative medical therapy using antibiotics and corticosteroids, some patients with medically refractory CRS require functional endoscopic sinus surgery [7,12]. However, synechiae/adhesions and post-operative stenosis are the most common and troublesome complications following FESS [63,66]. Nasal implants can be used clinically as adjuncts to endoscopic sinus surgery. They are primarily used to control hemorrhage, but also help to prevent adhesions formation [67] and promote drainage of the sinus mucosa, thereby promoting wound healing [68]. Drug-eluting nasal implants have been reported to reduce the rate of synechiae and stenosis formation. As inflammation/polyp recurrence, adhesions, and middle turbinate lateralization following FESS are very common outcomes; drug-eluting sinus implants can be used very effectively as adjuncts to sinus surgery because of their ability to preserve sinus patency by providing controlled, consistent drug release over a period of time to the sinus mucosa [69]. Specific applications of these devices are discussed in the following sections.

### 4.1. Drug Delivery Applications of Middle Meatus Implants

The middle meatus can be implanted with a spacer, implant or a sponge that preferably remain in place and biodegrade releasing drug load (corticosteroid or antibiotic) over an extended period of time without causing any tissue damage [63]. Drug eluting nasal implants not only improves the coverage of nasal passages, but also keeps the middle meatus open following FESS [70,71]. They are intended to fill the ethmoid sinus cavity, which would otherwise be filled with blood and mucous [63]. Examples of clinically approved middle meatal implants include Propel™ implant [72], Relieva Stratus™ MicroFlow spacer [73] and the Sinu-Foam™ spacer [74,75].

#### 4.1.1. Propel™ Sinus Implant

**Figure 5:**
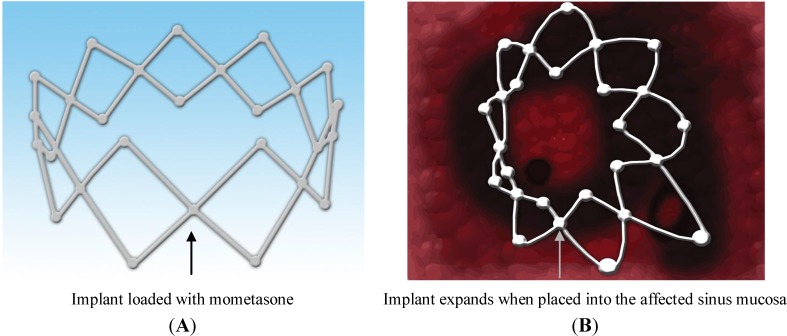
Mometasone-loaded (**A**) spring-like Propel™ sinus implant expands when placed into the sinus mucosa (**B**), thus keeping the middle meatus open and hence, promoting mucous drainage and wound healing.

Recently in 2011, Propel™ sinus implant was approved by the US Food and Drugs Administration (FDA) to be used clinically for the treatment of CRS. It is a mometasone-eluting biodegradable implant that continuously releases the drug locally in a controlled manner and for a prolonged period of time [72]. Propel™ is a spring like device placed in the ethmoid sinus cavity (Figure 5) by the physician as an adjunct to FESS to prevent postoperative obstruction of the cavity, which can occur due to inflammation and scarring [72]. The drug-eluting material in propel™ is biodegradable (PLGA). If this implant is placed in the affected sinus cavity, it dissolves (biodegrades) releasing the potent corticosteroid (mometasone), which is embedded in the PLGA matrix. Propel™ implant initiates a new era in the area of topical drug delivery for targeting the affected sinus mucosa providing controlled drug delivery directly to the sinus tissue [72]. A smaller version of the drug delivery system, propel™ mini is available for ease and convenience. It offers the same drug dose and clinical benefits, but varies in diameter (4.0 mm for propel mini *vs.* 5.2 mm for propel) and nominal expanded length (16 mm for propel mini *vs.* 23 mm for propel) from the standard system [76]. Propel mini is also an FDA-approved implant [77]. Propel dissolvable sinus implant has been clinically proven to prevent obstruction of the ethmoid sinus following surgery. It provides improved post-operative outcomes, reduces the need for additional surgery to remove the implant and the need for systemic steroids which can have serious side effects. Several clinical studies have been conducted to determine the safety and efficacy of this implant, when used in adult patients with CRS undergoing FESS. The ADVANCE II clinical trial, ADVANCE clinical trial and CONSENSUS II pilot study were conducted to determine its safety and efficacy. These trials were conducted in the United State with a total of 205 patients. A total of 400 implants were studied, of which 250 were drug-eluting and 150 were non-eluting implants used as control. Overall incidence rate of product related adverse events was about 1.5%, which included headache and recurrent sinusitis, which were resolved without sequelae. However, no patients withdrew due to an adverse event and no deaths were reported in any of the trials [78]. These clinical trials demonstrated the safety, efficacy and clinical utility of this biodegradable implant for the treatment of CRS [79]. Data from meta-analysis of patients enrolled in ADVANCE II and initial pilot study revealed that the use propel implant yielded 35% reduction in post-operative medical and surgical intervention, 40% reduction in the need for oral steroids and a 46% reduction in the rate of polyposis. The meta-analysis represents first level 1a evidence demonstrating the benefit of localized steroid delivery in the post-ESS period [80]. However, presently propel implant is approved for use only in the USA [72].

#### 4.1.2. Relieva Stratus™ Microflow Spacer

**Figure 6:**
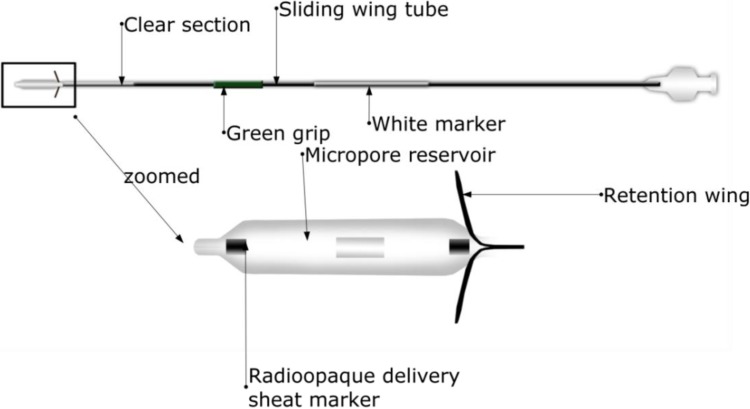
A typical design of a Relieva Stratus™ Microflow spacer, which contains a microporous reservoir, thus slowly releasing the drug to promote the wound healing of chronic ethmoid sinus mucosal disease.

Acclarent (Menlo Park, CA, USA) introduced Relieva Stratus™ for the treatment of sinusitis [81]. Relieva Stratus™ is a drug-eluting nasal implant for the treatment of chronic ethmoid sinus mucosal disease. This device has mainly two components: the deployment guide and MicroFlow spacer. The design and components of Relieva Stratus™ Microflow spacer are shown in Figure 6 (deployment guide not shown). Basically, MicroFlow spacer is a membrane reservoir surrounding a catheter shaft. The reservoir has several microspores that allow slow release of the instilled therapeutic agent into the target area. The device elutes the therapeutic agent slowly, continuously and in a controlled-manner for prolonged period of time. Relieva stratus™ is described as a minimally invasive option for the treatment of chronic ethmoid mucosal disease. It has been demonstrated that the device emits the steroid triamcinolone acetate for about 2–4 weeks after which it is removed in the office setting [73,81]. Certain disadvantages are associated with this device. It has been considered to be a leaky balloon instead of a conventional implant because of its fast drug release [81,82]. This device is temporary requiring manual removal after 30 days and a new device may be implanted if needed [83]. This device also is not suitable for patients with extensive polyps [73]. Finally, certain adverse events have been reported for the device [81] and orbital violation following its placement has been reported [84]. Although Relieva stratus™ can potentially be used with any therapeutic agent, it is currently approved by the FDA for use only with saline. This device is not intended for use with active drug substances as the safety and effectiveness of this device has not been demonstrated with an active drug substance in the reservoir. Use of steroids might result in high local and/or systemic concentrations, which may lead to serious adverse events [85]. This device is no longer marketed in the United States [83].

#### 4.1.3. Sinu-Foam™ Spacer

Sinufoam is an FDA approved mixture, which is commonly mixed with saline and gently placed in the ethmoid cavity following FESS [74]. A dexamethasone Sinu-Foam™ spacer was evaluated following FESS for CRS without polyps [75]. The spacer was found to promote wound healing of the nasal and sinus mucosa by reducing the inflammation associated with CRS [74]. However, its clinical utility remains a debate since it does not improve endoscopic outcomes in the early postoperative period following FESS [75]. It is not yet approved for use with steroids.

Other middle meatal stents have also been described in which a silicone-based stent can be used which comprises of two flanges, one of which separates the middle turbinate from the lateral nasal wall and one smaller flange securing the stent in the maxillary sinus [63,86]. However, silicone based-implant has a major disadvantage because it is not biodegradable and needs additional surgery for its removal. It has been reported that foams made of biodegradable synthetic polyurethane can also be used for patients that cannot tolerate other types of middle meatal stents [63]. However, clinical benefits of such delivery systems have not been demonstrated.

## 5. Challenges and Future Considerations

In spite of many advantages of drug-eluting nasal implants for CRS, many challenges still remain. As the implants are foreign materials implanted in the body, they can be associated with toxic shock syndrome (TSS). TSS can be associated with any retained foreign body (especially if it is not biodegradable). TSS has been reported in patients with frontal sinus stents [63,68,87]. The frontal sinus stents appeared to maintain sinus patency, but, were also associated with complications as noted in clinical studies, which included implant blockage and granulation build-up [81]. The variety of implants (Propel™ sinus implant, Relieva Stratus™ MicroFlow spacer and the Sinu-Foam™ spacer) that have been employed clinically as adjuncts to FESS show varying degrees of success. Data reported in literature showed significant variability in the outcome of these implants with respect to maintenance of sinus patency and drug release to the affected sinus mucosa [75]. Thus, further research needs to be conducted to demonstrate the efficacy of some of the implants.

The major disadvantage of Relieva Stratus™ MicroFlow spacer is its duration of use, 14–28 days (FDA clearance is for 14 days implantation) [73]. This is not sufficient to treat chronic inflammation of sinusitis, which lasts for more than 12 weeks [16] and thus a relapse of infection/inflammation can ensue. Also, it is currently FDA-approved for use only with saline and requires additional surgery for its removal which makes it unacceptable to most patients [88].

Propel™ sinus implant is the latest innovation in nasal implant research and has many advantages [89] over other available devices (Table 1). However, it is not approved for use outside the USA. Propel™ cannot be used in patients with intolerance to mometasone furoate [78]. Thus, there are limited options available for physicians and patients due to certain restrictions and individual patient to patient variability. Suggestions, comments and feedbacks from various doctors and physicians that have used drug-eluting implants for the treatment CRS can help overcome some of the drawbacks mentioned above. Although the development of propel™ sinus implant is an important break-through in the arena of drug-eluting biodegradable implants innovation, additional research is required to determine the appropriate steroid dose as the dose of mometasone in Propel™ is very low (370 µg) [72,78]. Patients in advanced stages of CRS with severe inflammation require higher doses of the drug which may lead to variability in results. More studies are needed to determine which patients are more likely to benefit from drug-eluting nasal implants.

**Table 1:** Comparison of the major advantages and limitations of Propel™ sinus implant, Relieva stratus™ Microflow spacer and Sinu-Foam™ spacer.

FDA Approved Nasal Stents/Implants	Advantages	Limitations
Propel™ sinus implant	Reduces inflammation associated with CRS and promotes wound healing; Implant is made of PLGA (a biodegradable polymer) and is the first and the only FDA approved biodegradable implant for the treatment of CRS; Does not require a second surgical procedure to remove the implant; Releases the drug slowly and continuously for over a month	Not approved for use outside the USA; Not suitable for patients with intolerance to mometasone furoate; Mometasone dose is quite low (370 µg) and may be ineffective in patients with advanced stages of CRS
Relieva stratus™ MicroFlow spacer	Potential to reduce chronic inflammation. Minimal invasive approach for targeted local delivery of therapeutic agents slowly and continuously to the site of action	Can be implanted only for 14–28 days; Requires a second surgical procedure for implant removal; FDA-approved for use only with saline
Sinu-Foam™ spacer	Promotes wound healing and reduces chronic inflammation of sinus and nasal mucosa	Clinical utility remains in doubt due to variable outcomes

## 6. Conclusions

Drug-eluting biodegradable nasal implants are promising next generation implants with considerable advantages. The biodegradable nature of these implants has eliminated most of the problems associated with metallic and other types of implants. Nevertheless, additional research is required to provide sufficient data to show their clinical efficacy and outcomes. The decision whether to use nasal implant or not completely depends on the individual surgeon and the condition of the patient. By overcoming certain limitations and conducting additional research in nasal implant development and optimization—particularly polymer selection, type and dose of drug candidates—nasal implants can become a major treatment option for CRS in the near future.
